# Modeling inpatient and outpatient antibiotic stewardship interventions to reduce the burden of *Clostridioides difficile* infection in a regional healthcare network

**DOI:** 10.1371/journal.pone.0234031

**Published:** 2020-06-11

**Authors:** Sarah Rhea, Kasey Jones, Stacy Endres-Dighe, Breda Munoz, David J. Weber, Rainer Hilscher, Jennifer MacFarquhar, Emily Sickbert-Bennett, Lauren DiBiase, Ashley Marx, James Rineer, James Lewis, Georgiy Bobashev, for the CDC MInD-Healthcare Network

**Affiliations:** 1 RTI International, Research Triangle Park, North Carolina; 2 UNC Health Care, Chapel Hill, North Carolina; 3 North Carolina Department of Health and Human Services, Raleigh, North Carolina; 4 Career Epidemiology Field Officer Program, Division of State and Local Readiness, Center for Preparedness and Response, Centers for Disease Control and Prevention, Atlanta, Georgia; 5 UNC Eshelman School of Pharmacy, Chapel Hill, North Carolina; ISI Foundation, ITALY

## Abstract

Antibiotic exposure can lead to unintended outcomes, including drug-drug interactions, adverse drug events, and healthcare-associated infections like *Clostridioides difficile* infection (CDI). Improving antibiotic use is critical to reduce an individual’s CDI risk. Antibiotic stewardship initiatives can reduce inappropriate antibiotic prescribing (e.g., unnecessary antibiotic prescribing, inappropriate antibiotic selection), impacting both hospital (healthcare)-onset (HO)-CDI and community-associated (CA)-CDI. Previous computational and mathematical modeling studies have demonstrated a reduction in CDI incidence associated with antibiotic stewardship initiatives in hospital settings. Although the impact of antibiotic stewardship initiatives in long-term care facilities (LTCFs), including nursing homes, and in outpatient settings have been documented, the effects of specific interventions on CDI incidence are not well understood. We examined the relative effectiveness of antibiotic stewardship interventions on CDI incidence using a geospatially explicit agent-based model of a regional healthcare network in North Carolina. We simulated reductions in unnecessary antibiotic prescribing and inappropriate antibiotic selection with intervention scenarios at individual and network healthcare facilities, including short-term acute care hospitals (STACHs), nursing homes, and outpatient locations. Modeled antibiotic prescription rates were calculated using patient-level data on antibiotic length of therapy for the 10 modeled network STACHs. By simulating a 30% reduction in antibiotics prescribed across all inpatient and outpatient locations, we found the greatest reductions on network CDI incidence among tested scenarios, namely a 17% decrease in HO-CDI incidence and 7% decrease in CA-CDI. Among intervention scenarios of reducing inappropriate antibiotic selection, we found a greater impact on network CDI incidence when modeling this reduction in nursing homes alone compared to the same intervention in STACHs alone. These results support the potential importance of LTCF and outpatient antibiotic stewardship efforts on network CDI burden and add to the evidence that a coordinated approach to antibiotic stewardship across multiple facilities, including inpatient and outpatient settings, within a regional healthcare network could be an effective strategy to reduce network CDI burden.

## 1. Introduction

Since 1978 when *Clostridioides difficile* (*C*. *difficile*) was first recognized as a cause of antibiotic-associated diarrhea and pseudomembranous colitis, *C*. *difficile* infection (CDI) has become the most common healthcare-associated infection (HAI) in the United States and is costly to the healthcare system [1]. Nonmodifiable CDI risk factors, like patient age and comorbidities (e.g., organ transplant, chronic renal disease, inflammatory bowel disease) have been described elsewhere [2]. However, a focus of prevention is to address established, modifiable CDI risk factors, specifically, exposure to antibiotics [2].

Exposure to antibiotics is associated with an increased risk of CDI [3]. Different antibiotics are associated with different levels of CDI risk. A meta-analysis quantified the risk of developing CDI in hospitalized patients receiving particular classes of antibiotics compared to those receiving no antibiotics [3]. Results from this meta-analysis support classification of clindamycin (associated CDI odds ratio (OR) = 16.8, 95% confidence interval (CI) 7.5 to 37.8), carbapenems and any cephalosporin (OR = 5.7, 95% CI 2.1 to 15.2), and fluroquinolones (OR = 5.5, 95% CI 4.3 to 7.1) as “high risk” CDI antibiotics, while penicillin combinations (OR = 2.7, 95% CI 1.8 to 4.2)  and trimethoprim/sulfonamides (OR = 1.8, 95% CI 1.3 to 2.4) can be classified as “moderate risk” CDI antibiotics [3]. Other antibiotics are considered lower risk for CDI [4]. The types of antibiotics prescribed and, therefore, the relative proportions of high-risk, moderate-risk, and low-risk antibiotics prescribed, can vary across healthcare settings (i.e., inpatient settings versus outpatient settings).

Improving antibiotic use is critical, not only to reduce an individual patient’s CDI risk, but also to mitigate emerging antibiotic resistance [5]. In U.S. acute care hospitals, 30%–50% of all prescribed antibiotics are considered inappropriate [6–11]. Inappropriate antibiotic prescribing can be defined as (1) unnecessary antibiotic prescribing (e.g., prescribing an antibiotic for a viral infection); (2) inappropriate antibiotic selection (e.g., prescribing an antibiotic associated with a higher risk of CDI when a lower risk antibiotic would be as or more effective); (3) inappropriate dose or duration of therapy; or (4) inappropriate antibiotic route of administration [12].

Efforts to reduce inappropriate antibiotic prescribing include antibiotic stewardship initiatives [13]. Antibiotic stewardship refers to a set of coordinated strategies designed to enhance patient health outcomes, reduce resistance to antibiotics, and decrease unnecessary costs [14]. Historically, antibiotic stewardship initiatives have focused on inpatient settings, especially within short-term acute care hospitals (STACHs). However, these efforts are expanding to long-term care facilities (LTCFs) (i.e., nursing homes and long-term acute care hospitals (LTACHs)) and outpatient settings in the community [13]. Antibiotic stewardship initiatives aimed at reducing inappropriate antibiotic selection, and restricting exposure to CDI high-risk antibiotics when lower risk antibiotics are appropriate, have proven effective in preventing CDI within STACHs [15]. Although emerging evidence documents the impact of antibiotic stewardship initiatives in LTCFs and in outpatient settings, the effects of specific interventions in these settings on CDI incidence are not well understood [16, 17].

The overall burden and transmission dynamics of *C*. *difficile* may be influenced by community sources and person movement among healthcare facilities [18–20]. Given this interconnectedness, a coordinated approach to antibiotic stewardship (i.e., implementation of interventions at multiple facilities, rather than at individual facilities) across a regional healthcare network may be most effective in reducing the overall burden of CDI in a region.

Agent-based models (ABMs) can capture the complexities of person movement among healthcare facilities and the communities they serve. Increasingly, ABMs are used to assess HAI prevention interventions, including antibiotic stewardship, which can inform decision making in public health and healthcare settings. Previous ABMs have demonstrated the reduction of CDI incidence associated with antibiotic stewardship initiatives in inpatient settings [21] and the benefits of a coordinated approach to interventions across inpatient settings in reducing incidence of CDI and other HAIs [22]. However, ABMs can also be used to estimate the impact of antibiotic stewardship efforts in LTCFs and outpatient settings and the effect of a coordinated antibiotic stewardship approach across regional healthcare networks.

We developed a geospatially explicit ABM of a regional healthcare network in North Carolina (NC) capable of examining the relative effectiveness of antibiotic stewardship interventions on CDI incidence [23]. We simulated reductions in unnecessary antibiotic prescribing and inappropriate antibiotic selection with intervention scenarios at individual and network healthcare facilities, including STACHs, LTCFs, and outpatient settings. Antibiotic exposure assigned to agents located in the community was conceptualized as the agent being “prescribed” the antibiotic at an outpatient healthcare facility. We estimated the effect of these interventions on hospital (healthcare) onset (HO)- and community acquired (CA)-CDI incidence trends across multiple simulations and discuss how this information could be used to inform antibiotic stewardship efforts across a regional healthcare network.

## 2. Methods

This study was approved by the Institutional Review Boards at the University of North Carolina at Chapel Hill and RTI International.

### 2.1 Regional healthcare network ABM with a CDI disease model

We previously developed a geospatially explicit ABM to simulate patient movement within a major regional healthcare network in NC, UNC Health Care [23]. UNC Health Care provides care for over 975,000 patients annually and is one of the largest healthcare systems in NC, with a catchment area of 5.9 million people across 41 counties [24, 25]. The ABM includes an academic medical center (UNC Hospitals [929 total beds]) and 9 affiliate hospitals (ranging from 81 to 665 total beds) [26]. We used a baseline synthetic population of NC, constructed using documented methods and code, which serve as agents for the ABM (**S1 File**) [23]. With a synthetic population approach, our ABM is effectively tied to actual geographies and populations and extends beyond single hospitals to include entire systems of communities and their associated healthcare networks [23]. Healthcare networks are groupings of related hospitals and other healthcare facilities that may be based on geopolitical boundaries (e.g., states), catchment areas (i.e., communities from which patients are drawn), and organizational structures (e.g., academic-affiliated medical campuses) [23].

Agents in the ABM represent >10.2 million NC residents [24]. Each agent can move among STACHs, LTACHs, nursing homes, and the community. Agents located in the community node can be conceptualized to be anywhere in the community other than an STACH, LTACH, or nursing home (e.g., home, outpatient healthcare facility). The ABM has a 1-day timestep and 1-year time horizon.

We updated the published ABM [23] by explicitly implementing 544 locations (i.e., location nodes), as follows: 10 regional network UNC STACH nodes (i.e., UNC Hospitals and 9 affiliate hospitals) [26]; 102 non-UNC STACH nodes (i.e., all 102 licensed STACHs in NC not affiliated with UNC Health Care) [27]; 421 nursing home nodes (representing the 421 NC licensed nursing homes) [28]; 10 LTACH nodes (representing the 10 NC licensed LTACHs) [27]; and 1 community node. We parameterized agent movement among the 544 location nodes using (1) de-identified patient-level data (UNC Health Care admissions during July 1, 2016─June 30, 2017), including length of stay (LOS); [23]; (2) publicly-available aggregate hospital discharge data for NC [25, 29]; (3) published demographic characteristics of nursing home residents and LTACH patients [30, 31]; (4) LOS distributions for nursing home patients according to Centers for Medicare & Medicaid Services (CMS) 2016 national patient-level fee-for-service claims data for CMS beneficiaries (subsequently referred to as CMS 2016 data); and (5) publicly-available NC licensed healthcare facility characteristics (i.e., geographic location, capacity, occupancy) [27, 30–33], as described in **S1 File** (see *v*. *Process overview and scheduling*). We calibrated agent movement according to the CMS 2016 data using methods described previously [34] and in **S1 File** (see *viv*. *Model verification*, *validation*, *and calibration*).

**Fig 1:**
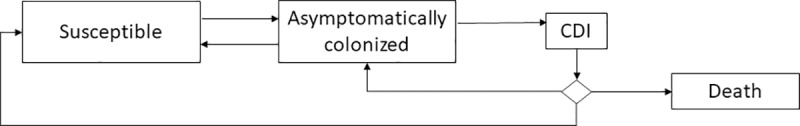
*Clostridioides difficile* natural history states (CDI: *Clostridioides difficile* infection).

We implemented a CDI disease model for each agent, with daily transition probabilities between disease states [23]. In the CDI disease model, agents exist in one of the following four disease states: susceptible, colonized, CDI, or dead (**Fig 1**). *C*. *difficile* asymptomatic colonization was a necessary step to develop CDI [35]. Transitions between disease states were dependent on an agent’s current location and other risk factors, including age, presence of comorbidities, and antibiotic exposure [23, 36–38] (**Table 1**). Transition to colonization was also dependent on the burden of colonization and CDI at the agent’s location (**S1 File**) [23, 38].

**Table 1:** Select^1^ *Clostridioides difficile* disease state and antibiotic parameter values.

Parameter	Assumed value(s)^2^	Reference
CDI transmission rate by agent location (i.e., node location in ABM)	STACH and LTACH: 2.1x10^-4^	*23*
	Nursing home: 8.6x10^-5^	
	Community: 6.3x10^-6^	
Antibiotic prescribing rates for non-network STACHs, LTACHs, nursing homes, and outpatient locations^3^^,^^4^	Non-network STACH: 0.37	*23*
	LTACH: 0.37	
	Nursing home: 0.005	
	Outpatient, <50 years of age: 1.3x10^3^	
	Outpatient, 50–64 years of age: 1.4x10^3^	
	Outpatient, ≥65 years of age: 1.7x10^3^	
Antibiotic course	10 days (SD = 2 days)	Expert opinion
Antibiotic risk ratios	Low risk: 2	*3*, *45*
	Moderate risk: 5	
	High risk: 12	
Baseline relative proportion of antibiotic use by risk class and location^3^^,^^4^	STACHs and LTACHs: proportion low risk = 0.4, proportion moderate risk = 0.3, proportion high risk = 0.3.	Calculated using patient-level data; *45*
	Nursing homes and outpatient locations: proportion low risk = 0.1, proportion moderate risk = 0.6, proportion high risk = 0.3	

ABM: Agent-based model; LTACH: long-term acute care hospital; SD: standard deviation; STACH: short-term acute care hospital.

^1^See Appendix for additional parameter values.

^2^Assumed value rates are per day.

^3^Antibiotic exposure assigned to agents located in STACHs, LTACH, or nursing home nodes was conceptualized as the agent being “prescribed” the antibiotic at that healthcare facility.

^4^Antibiotic exposure assigned to agents located in the community node was conceptualized as the agent being “prescribed” the antibiotic at an outpatient location.

### 2.2 Modeling antibiotic exposure

Each agent in the ABM existed in a dynamic, binary state of antibiotic exposure (i.e., with antibiotic exposure [during the antibiotic course and for 90 days following the completion of the course] or without antibiotic exposure). Daily probabilities of antibiotic exposure were informed by agent location and age [38]. Antibiotic exposure assigned to agents located in STACH, LTACH, or nursing home nodes was conceptualized as the agent being “prescribed” the antibiotic at that healthcare facility. Antibiotic exposure assigned to agents located in the community node was conceptualized as the agent being “prescribed” the antibiotic at an outpatient healthcare facility.

We used patient-level data to calculate antibiotic prescribing rates and length of therapy (LOT) per 1,000 patient-days in each of the seven network STACHs for which patient-level data were available from UNC Health Care [39, 40]. LOT was selected as the antibiotic consumption metric for this study, as it represents days of antibiotic exposure without regard to the number of antibiotics used. Specifically, we defined LOT as the number of days that a patient received systemic antibiotics during their admission (i.e., duration of antibiotic use), regardless of the number of antibiotics received. For each of the three network STACHs for which patient-level data were not available, we assigned the mean LOT value of the network STACH(s) in the same intensive care unit bed category [41, 42]. We selected categorization by intensive care unit bed size, as it is commonly employed for HAI public health surveillance activities [41, 42].

When an agent was assigned to antibiotic exposure, this assignment included (1) an antibiotic course duration and (2) an antibiotic risk level (i.e., low-, moderate-, or high-risk antibiotic) (**Table 1**). Agents with antibiotic exposure were at increased risk of CDI according to static risk ratios (RRs) associated with each antibiotic risk level, selected to simulate varied risk corresponding to different antibiotic classes, during the antibiotic course and for 90 days following the completion of the course [3, 4, 43, 44]. We used patient-level data from UNC Health Care to inform the relative antibiotic risk level proportions applied to each location; each location’s proportion of low-, moderate-, and high-risk antibiotics prescribed summed to one [38, 45]. Each agent’s antibiotic-associated risk of CDI exponentially decreased during day 30 to day 90. No antibiotic-associated CDI risk was modeled by day 91 following completion of antibiotic course (**S1 File**).

An agent could only be assigned a new course of antibiotics once it completed an existing course. However, a new course could be initiated at any other time, including during the 90-day residual risk period. If an agent received a subsequent antibiotic assignment during the residual risk period of a previously completed antibiotic course, the agent was assigned the higher of the two possible antibiotic risk levels for the antibiotic course duration.

We calibrated the ABM to reproduce both colonization prevalence rates and antibiotic exposure rates by location [23, 38, 46] (**S1 File**). Subsequently, we calibrated the ABM to reproduce the following CDI incidence targets: (1) HO-CDI according to NC-specific National Healthcare Surveillance Network *C*. *difficile* lab event data by hospital; (2) published community onset (CO)-CDI incidence [47–49]; (3) published CA-CDI incidence [50–53]; and (4) published healthcare associated (HA)-CDI incidence [50–53]. We assigned a small number of agents to transition CDI within their first 3 days of admission to a healthcare facility to meet CO-CDI incidence calibration targets (**S1 File**). We used established CDI case definitions, adapted to the context of this ABM, with each CDI case defined (1) as either CO-CDI or HO-CDI and (2) as either CA-CDI or HA-CDI (**S1 File**). Specifically, we defined a CDI case as CO-CDI if the agent transitioned to the CDI disease state fewer than 3 days after admission to a healthcare facility (STACH, LTACH, nursing home), and HO-CDI if the agent transitioned to the CDI disease state at least 3 days after admission to a healthcare facility (STACH, LTACH, nursing home) [47]. Additionally, we defined a CDI case as CA-CDI if the agent transitioned to the CDI disease state while in the community node or within 3 days after admission to a healthcare facility (STACH, LTACH, nursing home) and the agent had not been admitted to a healthcare facility in the preceding 12 weeks; all CDI cases that did not meet these CA-CDI criteria were classified as HA-CDI [51, 52]. We considered CDI cases that transitioned to the CDI disease state between 2 and 8 weeks of the last CDI disease state transition to be recurrent episodes and CDI cases that transitioned to the CDI disease state less than 2 weeks since the last CDI disease state transition to be duplicate episodes; all others were considered incident cases [51, 52].

### 2.3 Antibiotic stewardship interventions

We modeled two antibiotic stewardship interventions, each representing a decrease in inappropriate antibiotic use, as follows: (1) reduction in total antibiotics prescribed at individual facility types alone and across all network facilities (i.e., coordinated stewardship approach) (five scenarios total); (2) reduction in CDI high-risk antibiotics, in favor of CDI moderate-risk antibiotics, prescribed at individual facility types (two scenarios total).

#### 2.3.1. Reduction in total antibiotics prescribed

We developed five scenarios exploring the impact of a reduction in antibiotics prescribed at individual healthcare facility types and across healthcare facility types, representing a coordinated stewardship approach, as follows: (1) network STACHs only; (2) nursing homes only; (3) outpatient facilities only; (4) all network STACHs and nursing homes; and (5) all network inpatient and outpatient facilities (i.e., STACHs, nursing homes, LTACHs, and outpatient locations). For each scenario, the total antibiotics prescribed were reduced by 10%, 20%, and 30%. Of note, across the network the absolute decrease in the number of antibiotics prescribed differed with each of these scenarios. For example, a 10% decrease in total antibiotics prescribed in STACHs and a 10% decrease in total antibiotics prescribed in nursing homes are not equal in terms of the number of antibiotic doses averted. We selected a 30% reduction as the maximum reduction possible in our simulated interventions, as at least 30% of antibiotics prescribed in outpatient setting in the United States are reportedly inappropriate [54].

#### 2.3.2 Reduction in high-risk antibiotics prescribed

We developed two scenarios exploring the impact of a reduction in high-risk antibiotics prescribed at individual facility types: (1) network STACHs, and (2) nursing homes. For each scenario, the relative proportion of CDI high-risk antibiotics was reduced by 0.1, with a concordant 0.1 increase in moderate-risk antibiotics prescribed, and by 0.2, with a concordant 0.2 increase in moderate-risk antibiotics prescribed.

We conducted 40 runs for each scenario using the entire NC synthetic population (>10.2 million agents) as microdata input to the ABM to account for the complex movement of agents throughout the state. We defined the following model outcomes for the 41-county catchment area of the network [23] to assess the relative effectiveness of each intervention, as follows: HO-CDI incidence per 10,000 patient-days in network STACHs (i.e., the 10 STACHs of UNC Health Care), HO-CDI incidence per 10,000 patient-days in nursing homes, HO-CDI incidence per 10,000 patient-days in network inpatient healthcare facilities (i.e., 10 STACHs of UNC Health Care, nursing homes, and LTACHs), and CA-CDI incidence per 100,000 population. Incidence measures are presented as a mean, with range based on standard deviation and 95% CIs, of the multiple model runs. We calculated percent change in CDI incidence to compare each intervention to baseline (i.e., no intervention), which corresponds to the data that were used to design the ABM and reflects established antibiotic prescribing.

## 3. Results

### 3.1 Antibiotic prescribing rates in network STACHs

Antibiotic prescribing rates among admissions to the seven UNC STACHs for which data existed (i.e., Hospitals 1–7) ranged from 251 LOT per 1,000 patient-days to 450 LOT per 1,000 patient-days (**Table 2**).

**Table 2:** Antibiotic prescribing rates and length of therapy (LOT) per 1,000 patient-days among admissions to network short-term acute care hospitals (STACHs).

STACH^1^	Number of intensive care unit beds (range)^2^	Antibiotic prescribing rate	LOT per 1,000 patient days
Hospital 1	10–19	0.45	450
Hospital 2	5–9	0.33	331
Hospital 3	20–42	0.25	251
Hospital 4	10–19	0.33	331
Hospital 5	5–9	0.33	332
Hospital 6	≥43	0.28	281
Hospital 7	≥43	0.29	290
Hospital 8	20–42	0.25^3^	251^3^
Hospital 9	10–19	0.39^4^	390^4^
Hospital 10	10–19	0.39^4^	390^4^

^1^UNC Health Care STACH names are masked per data use agreement.

^2^Based on categories from following resource: N.C. Communicable Disease Branch. Healthcare-associated infections in North Carolina. Reporting period: January 1–June 30, 2016. 2016 [cited July 31, 2019]. Raleigh, NC: N.C. Surveillance for Healthcare-Associated and Resistant Pathogens Patient Safety (SHARPPS) Program. Available at https://epi.dph.ncdhhs.gov/cd/hai/figures/2016/2016Q2_Hospital_Specific_Quarterly_Report.pdf

^3^Used Hospital 3 daily rate.

^4^Used mean of Hospitals 1 and 4 daily rates.

### 3.2 Reduction in total number of antibiotics prescribed

**Fig 2:**
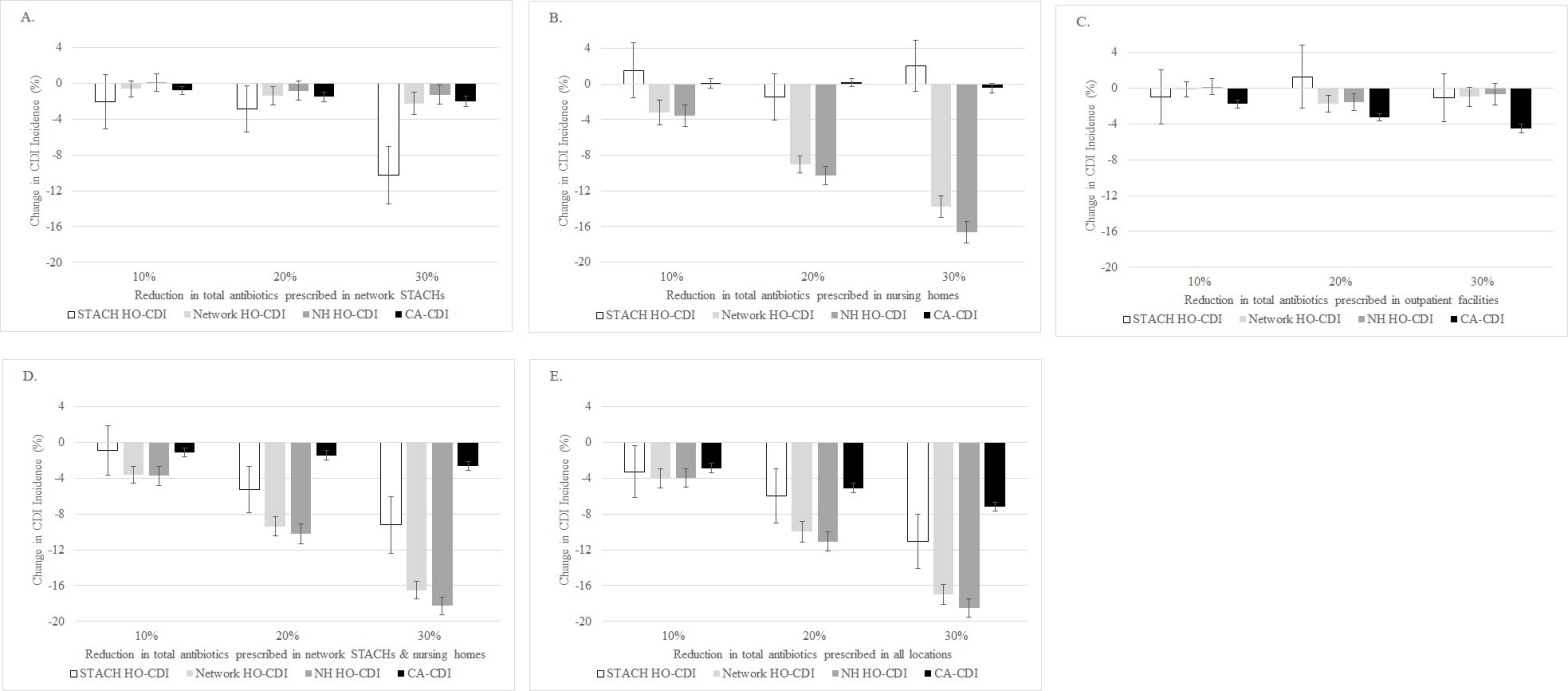
*Clostridioides difficile* infection (CDI) incidence and reductions in total number of antibiotics prescribed. Scenarios for reduction in total number of antibiotics prescribed are as follows: (A) at network STACHs only; (B) at nursing homes only; (C) at outpatient facilities only; (D) at all network STACHs and nursing homes; and (E) at all network inpatient (i.e., STACHs, nursing homes, LTACHs) and outpatient facilities. For each scenario, the total antibiotics prescribed were reduced by 10%, 20%, and 30%. Results are based on 40 runs for each scenario. Error bars indicate 95% confidence intervals on the percent change. (STACH: short-term acute care hospital; LTACH: long-term acute care hospital; HO-CDI: hospital (healthcare) onset-CDI; CA-CDI: community associated-CDI).

As the total number of antibiotics prescribed within network STACHs and nursing homes decreased by 10%, 20%, and 30%, the HO-CDI incidence in the ABM also decreased within that location category (**Fig 2A and 2B**). Specifically, as antibiotics prescribed in STACHs decreased by 30%, the STACH HO-CDI incidence decreased by 10%, from 7.0 cases per 10,000 patient-days (95% CI 6.3–7.7 cases per 10,000 patient-days) to 6.3 cases per 10,000 patient-days (95% CI 5.6–7.0 cases per 10,000 patient-days) (**Fig 2A**). As the antibiotics prescribed in nursing homes decreased by 30%, the nursing home HO-CDI incidence decreased by 17%, from 8.4 cases per 10,000 patient-days (95% CI 7.1–9.8 cases per 10,000 patient-days) to 7.0 cases per 10,000 patient-days (95% CI 5.9–8.2 cases per 10,000 patient-days) (**Fig 2B**).

As the total number of antibiotics prescribed in STACHs decreased by 30%, the network HO-CDI incidence decreased by 2%, from 8.6 cases per 10,000 patient-days (95% CI 7.3–9.8 cases per 10,000 patient-days) to 8.4 cases per 10,000 patient-days (95% CI 7.2–9.6 cases per 10,000 patient-days) (**Fig 2A**). Similarly, as the antibiotics prescribed in nursing homes decreased by 30%, network HO-CDI incidence decreased by 14%, to 7.4 cases per 10,000 patient-days (95% CI 6.3–8.4 cases per 10,000 patient-days) (**Fig 2B**). With a reduction in outpatient antibiotics only, we observed a minimal reduction (1%) in CDI incidence at other network locations (**Fig 2C**).

With a coordinated stewardship approach across all network STACHs and nursing homes in the ABM, a 30% reduction in antibiotics prescribed resulted in a 17% decrease in network HO-CDI incidence, to 7.1 cases per 10,000 patient-days (95% CI 6.1–8.1 cases per 10,000 patient-days) and a 3% decrease in CA-CDI incidence, from 122.8 cases per 100,000 person-years (95% CI 108.8–136.8 per 100,000 person-years) to 119.6 cases per 100,000 person-years (95% CI 105.9–133.2 per 100,000 person-years) (**Fig 2D**). With a coordinated stewardship approach across all network inpatient and outpatient locations, reducing the antibiotics prescribed by 30%, there was a 17% decrease in network HO-CDI incidence, to 7.1 cases per 10,000 patient-days (95% CI 6.1–8.1 cases per 10,000 patient-days) and a 7% decrease in CA-CDI incidence, to 114.2 cases per 100,000 person-years (95% CI 101.0–127.3 cases per 100,000 person-years) in the ABM (**Fig 2E)**.

With this intervention scenario, we observed occasional increases in CDI incidence. Specifically, we observed a 0.1% increase in nursing home HO-CDI incidence with a 10% reduction in total antibiotics prescribed in network STACHs (**Fig 2A**). With 10% and 30% reductions in total antibiotics prescribed in nursing homes, we observed 2% increases in STACH HO-CDI incidence (**Fig 2B**). We observed a 1% increase in STACH HO-CDI incidence with a 20% reduction in total antibiotics prescribed in outpatient facilities (**Fig 2C**).

### 3.3 Reduction in antibiotic risk

**Fig 3:**
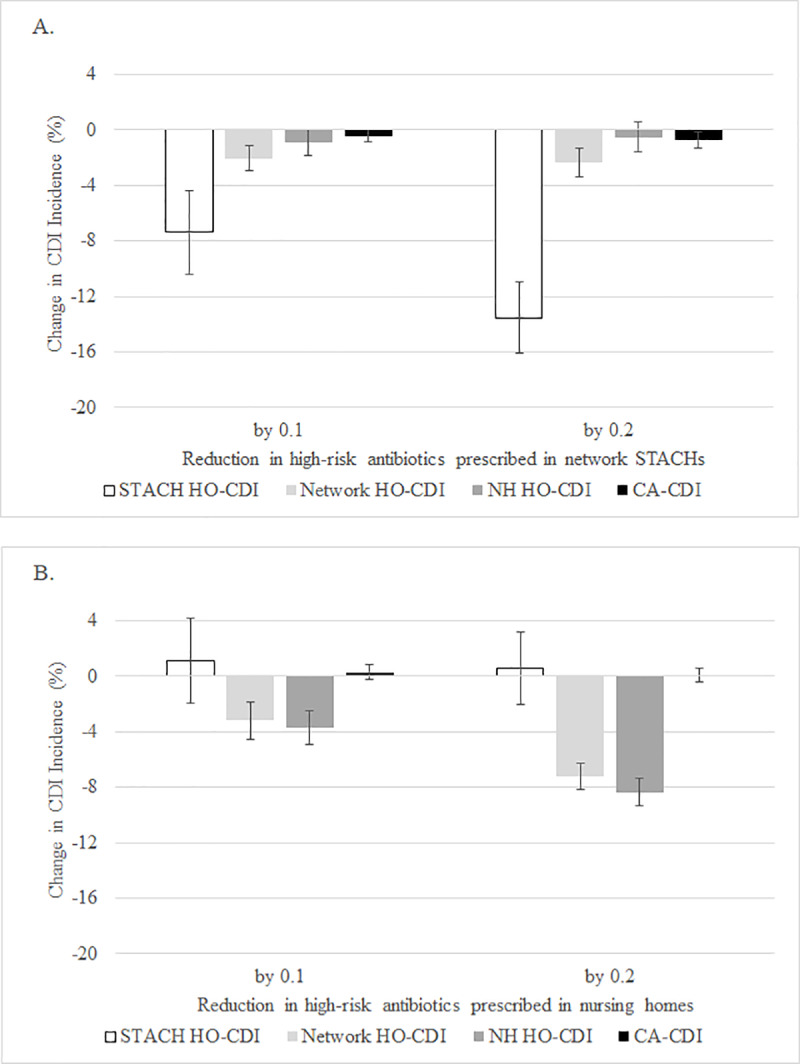
*Clostridioides difficile* infection (CDI) incidence and reductions in high-risk antibiotics prescribed. Scenarios as follows: (A) at network STACHs only; and (B) at nursing homes only. For each scenario, the relative proportion of CDI high-risk antibiotics was reduced by 0.1, in favor of a 0.1 increase in moderate-risk antibiotics prescribed, and by 0.2, in favor of a 0.2 increase in moderate-risk antibiotics prescribed. Results are based on 40 runs for each scenario. Error bars indicate 95% confidence intervals on the percent change. (STACH: short-term acute care hospital; HO-CDI: hospital (healthcare) onset-CDI; CA-CDI: community associated-CDI).

As the relative proportion of high-risk antibiotics prescribed in STACHs decreased by 0.2 (in favor of moderate-risk antibiotics), the STACH HO-CDI incidence decreased by 14%, to 6.1 cases per 10,000 patient-days (95% CI 5.4–6.6 cases per 10,000 patient-days), and the network HO-CDI incidence decreased by 2%, to 8.4 cases per 10,000 patient-days (95% CI 7.2–9.6 cases per 10,000 patient-days) (**Fig 3A**). Similarly, as the relative proportion of high-risk antibiotics prescribed in nursing homes decreased by 0.2 (in favor of moderate-risk antibiotics), the nursing home HO-CDI incidence decreased by 8%, to 7.7 cases per 10,000 patient-days (95% CI 6.5–8.9 cases per 10,000 patient-days), and the network HO-CDI incidence decreased by 7%, to 7.9 cases per 10,000 patient-days (95% CI 6.8–9.1 cases per 10,000 patient-days) (**Fig 3B**). With a reduction in high-risk antibiotics prescribed in nursing homes, we observed a 1% increase in STACH HO-CDI incidence and a 0.3% increase in CA-CDI incidence (**Fig 3B**).

## 4. Discussion

We implemented antibiotic stewardship interventions in a geospatially explicit ABM of a regional healthcare network and examined the relative effectiveness of these interventions on CDI incidence compared to baseline. Our ABM demonstrated relative changes in CDI incidence based on simulated antibiotic stewardship initiatives to (1) reduce unnecessary antibiotics at individual facility types compared to a coordinated approach across network locations; and (2) reduce inappropriate antibiotic selection in STACHs and in nursing homes. Although modest, the general trends in CDI incidence reduction and the relationships between implementation at individual facility types and other network locations are apparent.

We observed a greater impact on network HO-CDI by simulating a reduction in antibiotic prescribing in nursing homes alone (14% HO-CDI reduction) compared to the same intervention in STACHs alone (2% HO-CDI reduction). This finding is notable considering that the absolute number of antibiotic courses prescribed in our ABM varies by healthcare facility type. During a typical run of the ABM, the number of antibiotic courses initiated in STACHs is approximately seven-times greater than the number of antibiotic courses initiated in nursing homes. Therefore, a 30% reduction in total antibiotics administered in network STACHs is a larger reduction in the absolute number of antibiotic doses than a 30% reduction in total antibiotics administered in nursing homes. However, the nursing home LOS (mean: 95 days) is longer than the STACH LOS (range of means: 4.3–6.6 days) in the ABM (**S1 File**) [23]. Agents in nursing homes are, therefore, at increased CDI risk for a longer time period compared to agents at STACHs. Additionally, these agents who are prescribed antibiotics will likely remain in a nursing home for most their antibiotic exposure time, increasing their CDI risk because of facility-based transmission rates (**S1 File**) compared to agents discharged to their homes. Finally, agents in nursing homes are inherently at increased risk of CDI due to their age (≥65 years of age).

By simulating a reduction in inappropriate antibiotic selection, we estimated a relative reduction in CDI high-risk antibiotics prescribed, in favor of moderate-risk antibiotics, in STACHs and nursing homes. In these simulations, the interventions focused at nursing homes alone resulted in a larger impact on CDI incidence, compared to similar interventions at network STACHs alone. Again, this finding can largely be explained by the longer LOS for agents in nursing homes, compared to STACHs, in the ABM and their inherent increased risk of CDI due to age.

With each of the two antibiotic stewardship interventions modeled, we observed occasional simulation results of increased CDI incidence. For the intervention scenario of reducing total antibiotics prescribed, these observations were inconsistent across percent reductions (**Fig 2A, 2B and 2C**). For example, 10%, 20%, and 30% reductions in total antibiotics prescribed in nursing homes resulted in a 2% increase, a 2% decrease, and a 2% increase, respectively, in STACH HO-CDI incidence (**Fig 2B**). Similarly, the greater the reduction in high-risk antibiotics prescribed in nursing homes, the smaller the increase in STACH HO-CDI incidence (**Fig 3B**). This may be related to random variation during simulations. In the future, we will consider conducting additional simulation runs, beyond the 40 simulation runs presented here, to further investigate this variation.

Among our interventions for reduced antibiotic prescribing, we found a maximum impact on CDI incidence when modeling the intervention across all network inpatient and outpatient locations, with a 17% reduction in network HO-CDI incidence and a 7% reduction in CA-CDI incidence. These results support a coordinated approach to antibiotic stewardship across multiple facilities and facility types as an effective strategy to reduce the burden of CDI in regional healthcare networks [55, 56].

This modeling study is unique in considering outpatient prescribing, conceptualized as agents located in the community node being “prescribed” the antibiotic at an outpatient location. Healthcare facilities within a network are interconnected with each other and their catchment areas through person movement. By including the community in our ABM, this study brings an enhanced understanding of the potential impact of antibiotic stewardship interventions on CDI incidence in the community. This not only provides additional nuance to our interpretations but also makes the ABM more realistic, which could aid in translation of findings to public health and healthcare stakeholders, especially those who are unfamiliar with ABMs.

We calculated daily antibiotic prescribing rates at network STACHs using patient-level data from UNC Health Care, which varied slightly across facility intensive care unit bed categories. Although the impact of this variability is not established, future work could include evaluating the sensitivity of the model output to variable daily prescribing rates across different healthcare facility types (e.g., tertiary care versus critical access STACH). Further, these daily prescribing rates could be informed by data from other healthcare systems in other regions.

There are several limitations to the study. Our results suggest modest relative effects that make some scenario comparisons challenging to interpret. For example, a reduction in antibiotics prescribed at STACHs alone resulted in a 14% reduction in STACH HO-CDI incidence, while a reduction in antibiotics prescribed across all network locations produced only an 11% reduction in STACH HO-CDI incidence. This counterintuitive finding can be explained by the negligible difference between the actual HO-CDI incidence values, which are quite similar over the 40 model simulations (6.0 cases per 10,000 patient-days and 6.2 cases per 10,000 patient-days, respectively). This may also be related to random variation during simulations or to the antibiotic assignment rules that we employed (e.g., an agent could only be assigned a new course of antibiotics once it completed an existing course). In the future, we will consider conducting additional simulation runs, beyond the 40 simulation runs presented here, to further investigate this variation. Although community administration of antibiotics did not increase the likelihood that an agent was transferred to a healthcare facility, this update could be implemented in subsequent iterations of the ABM. Finally, other types of models (e.g., deterministic differential equation model) could arguably be used to evaluate antibiotic stewardship interventions as presented in this manuscript. However, we view these interventions and our results as initial steps in our effort to use this ABM to study a variety of simulated interventions as follow-on studies.

We employed the antibiotic stewardship interventions uniformly across all 10 network STACHs, but this does not impart the maximum possible variation that the ABM can provide across these 10 sites. Similarly, outpatient prescribing was treated uniformly across the community location node. However, variability in outpatient prescribing varies by geography and facility type [46]. We assumed that the interventions were implemented with perfect specificity, only reducing inappropriate antibiotic prescribing and not appropriate antibiotic prescribing.

We evaluated the two antibiotic stewardship interventions independently. Realistically, these interventions would be applied in coordination and to varying degrees across individual facilities and multiple facilities, as stewardship efforts should be directed toward the most relevant factors applicable to that setting [57]. Future simulations will address these limitations by accounting for variability in prescribing practices across locations and by exploring implementation of antibiotic stewardship interventions at single STACHs of different types (e.g., tertiary care, critical access) on incidence of *C*. *difficile* and other HAI pathogens. We conceptualized reduction of inappropriate antibiotic use as shifts from higher-risk to moderate-risk CDI antibiotics. The impact of successful antibiotic stewardship efforts at different facility types will lead to greater variation in proportions of low, moderate, and high-risk CDI antibiotics than was modeled in this study.

All antibiotic prescribers and healthcare facilities are responsible for antibiotic stewardship efforts. This responsibility includes a determination if antibiotics are necessary and, if so, the optimal antibiotic selection, dose, duration, and route [12]. Antibiotic stewardship efforts may be maximized if implemented across multiple healthcare facilities serving a population. Our findings build on the established modeling literature that supports the effectiveness of a coordinated approach for antibiotic stewardship across multiple facilities. This study also uniquely demonstrates the added impact of antibiotic stewardship interventions in LTCF and outpatient locations across a regional healthcare network for a reduction in the burden of HAIs.

## Supporting information

S1 File Overview, Design concepts, and Details (ODD). (DOCX)
