# Supplementary material for: Modeling inpatient and outpatient antibiotic stewardship interventions to reduce the burden of Clostridioides difficile infection in a regional healthcare network
Source: PLoS One. 2020 Jun 11;15(6):e0234031. doi: 10.1371/journal.pone.0234031 (PMC7289388; doi:10.1371/journal.pone.0234031)
Supplement: S1 File — (DOCX) [file pone.0234031.s001.docx]

**S1. File. Overview, Design Concepts, Details (ODD) Protocol**

The ODD protocol provides a standardized way of describing an agent-based model (ABM) so that other researchers can implement the model, promoting open science and replicability [1, 2]. Model source code and supporting data can be found at the following location: <https://github.com/RTIInternational/NCMInD>

***i. Purpose and scope.*** We use a geospatially explicit ABM with a *Clostridioides* *difficile* (*C. difficile*) infection (CDI) disease model derived from a previously published CDI compartmental model [3]. Our disease model is based on four key assertions regarding *C. difficile*. First, risk factors for CDI include the use of antibiotics, advanced age, exposure to inpatient healthcare settings, and underlying concurrent medical conditions [4, 5]. Second, individuals who are exposed to antibiotics have residual increased risk of CDI for at least 90 days following the end of treatment therapy [6, 7]. Third, *C. difficile* asymptomatic colonization, when a person tests positive for the *C. difficile* organism or its toxin but exhibits no clinical symptoms, is a necessary step to develop CDI [8]. Fourth, *C. difficile–*infected individuals are typically treated with one or more antibiotics during their infection and recovery [9].

***ii. Entities, state variables, and scales.*** The ABM has two types of entities: locations and agents. Locations are represented by 544 nodes in a geospatially explicit network that defines the movement of agents between these locations in North Carolina (NC). Location types are as follows: short-term acute care hospital (STACH), long-term acute care hospital (LTACH), nursing home, and community. STACH nodes include the following: 10 UNC Health Care (UNC) STACH nodes [10], 90 non-UNC nodes of STACHs with <400 beds (i.e., small non-UNC STACHs); 12 non-UNC nodes of STACHs with ≥400 beds (i.e., large non-UNC STACHs) [10-13]. Furthermore, we implemented 421 nursing home nodes, 10 LTACH nodes, and one community node [13, 14]. We do not model movement among households or between- or within-household infection dynamics. Agents located in the community node can be conceptualized to be anywhere in the community other than an STACH, LTACH, or nursing home (e.g., home, outpatient healthcare facility). Agents are defined by sex (female, male); age group in years (<50, 50−64, ≥65 years); race (white, black, other race); and NC home county of residence (100 NC counties). Agents can move among different locations and can change both disease and antibiotic-use states. The ABM is implemented with a 1-day time step and a 1-year time horizon. That is, agents may change each state only once per day. The variables that drive an agent’s location movement (age group and NC county of residence) are not updated during the 1-year model run. Comorbidities are assigned based on published proportions [3], and an agent’s probability of moving is informed by its comorbidity status. The presence of comorbidities increases the probability of an agent moving to an STACH from the community.

***iii. Submodels:*** The ABM has two submodels, a location model and a CDI disease model.

The ***location model*** defines agent movement on the location network. There are 544 possible locations (see ***ii. Entities, state variables, and scales***). Each agent can move to any other location, within a geographically constrained subset of possible locations, from its initialized location (**S1 File Figure 1**). The subset of possible locations is defined by the geographic home location (i.e., NC home county of residence) of an agent. When an agent is assigned to move to a healthcare facility of a certain category, the agent generally moves to a healthcare facility of that category that is in or near the agent’s NC home county of residence. For example, if an agent is assigned to move to a nursing home, that agent will preferentially move to a nursing home within its NC home county of residence. However, an agent can move to a healthcare facility outside of its home county under certain circumstances (e.g., agent is assigned to move to one of the 12 large non-UNC STACHs and does not have one of these STACHs in its home county).

We used aggregate hospital discharge data [11, 12] to develop discharge distributions to inform an agent’s initial STACH assignment (when selected to move to an STACH) by type (UNC STACH, small non-UNC STACH, large non-UNC STACH) and an agent’s movement among STACHs (when selected to be transferred). Patient county of residence by hospital discharge data only includes counties that account for ≥1% of discharges. When, because of censored values, the aggregate hospital discharge data cannot be used, we apply different restrictions for movement according to the healthcare facility type, as described in more detail below.

*Small (<400 beds) non-UNC STACHs:* Small non-UNC STACH discharge data were available for all but one NC county. The ABM uses distributions created from these available discharge data to randomly assign agents selected to move to a small non-UNC STACH. Agents that are discharged from a small non-UNC STACH can be selected to move to another small non-UNC STACH, with assignment based on the distributions. Agents are not permitted to remain at the same small non-UNC STACH once their length of stay (LOS) has expired. Rather, in this rare situation, if an agent is selected to transfer from a small non-UNC STACH to another small non-UNC STACH and that agent is from a county with discharge data available for only one small non-UNC STACH, the ABM randomly assigns the agent to another small non-UNC STACH for this transfer.

*Large (≥400 beds) non-UNC STACHs:* Large non-UNC STACH discharge data were available for all but 26 counties. The ABM uses distributions created from these available discharge data to randomly assign agents selected to move to a large non-UNC STACH. If an agent who is selected to move to a large non-UNC STACH is from a county with no large non-UNC STACH discharge data available, the ABM assigns the agent to the geographically closest large non-UNC STACH based on the agent’s U.S. Census block group (for home residence). Similarly, if an agent is selected to move from a large non-UNC STACH to another large non-UNC STACH, the ABM uses the distributions, followed by the block group distance, to assign the agent. In the rare situation that both these methods fail, the ABM randomly assigns the agent to a large non-UNC STACH.

*UNC STACHs*: Agent movement to UNC STACHs is based on the discharge data for each of the 10 modeled UNC STACHs which serve a 41-county catchment area. Most of the agent movement to and from UNC STACHs is completed by agents whose home county is among the 41-county catchment area. The ABM uses distributions that we created from the available discharge data to select an agent’s initial UNC STACH and inform its movement from one UNC STACH to the next UNC STACH (i.e., transfer). If an agent is selected to transfer from a UNC STACH to another UNC STACH and that agent is from a county with discharge data available for only one UNC STACH, the ABM randomly assigns the agent to one of the two largest UNC STACHs for this transfer.

When an agent arrives at an STACH or LTACH, the agent is assigned an LOS based on a gamma distribution unique to the healthcare facility. We use patient-level data, available for 7 of the 10 UNC STACHs, to obtain STACH-specific LOS gamma distributions. For the remaining three UNC STACHs and the non-UNC STACHs, for which patient-level data were not available, we used aggregate discharge data to estimate the parameters of a gamma distribution [12]. When an agent arrives at a nursing home, the agent is assigned an LOS based on a distribution created from the Centers for Medicare & Medicaid Services (CMS) 2016 national patient-level fee-for-service claims data for CMS beneficiaries.

Upon developing CDI an agent’s LOS is increased by 3 days [15, 16]. When an agent’s LOS is complete, the agent moves from the healthcare facility to another location. The agent cannot remain at the healthcare facility past its assigned LOS. When the agent moves to another healthcare facility, the agent is assigned a new LOS. We simulate agent movement based on the following agent characteristics: (1) current location, (2) NC home county of residence, (3) age group in years (<50, 50-64, ≥65), and (4) presence of comorbidities (yes, no) (**S1 File Table 1**).

A ***CDI disease model***, derived from Durham and colleagues’ CDI compartmental model, is implemented in the ABM [3]. In our disease model, agents can move between one of the following four disease states: (1) susceptible, (2) asymptomatically colonized, (3) CDI, and (4) death associated with CDI (**S1 File Figure 2, S1 File Table 2**). Each agent exists in a dynamic, binary state of antibiotic exposure (i.e., with or without antibiotic exposure). Daily probabilities of antibiotic exposure are informed by agent location and age. Antibiotic exposure assigned to agents located in STACHs, LTACHs, or nursing home nodes is conceptualized as the agent being “prescribed” the antibiotic at that healthcare facility. Antibiotic exposure assigned to agents located in the community node is conceptualized as the agent being “prescribed” the antibiotic at an outpatient healthcare facility.

When assigned to antibiotic exposure, this assignment includes (1) an antibiotic course duration and (2) an antibiotic risk level (i.e., low-, moderate-, or high-risk antibiotic) (**S1 File Tables 2 and 3**). Agents with antibiotic exposure are at increased risk of CDI according to static risk ratios (RRs) associated with each antibiotic risk level, selected to simulate varied risk corresponding to different antibiotic classes. Agents that reach the CDI state who have completed their original antibiotic course are subsequently assigned a new course of antibiotics.

The probability of receiving antibiotics depends on the current location of the agent, with agents in the community (i.e., those receiving outpatient antibiotics) having probabilities additionally informed by age. Antibiotic exposure duration is approximately 100 days. Agents receive an initial course of antibiotics from a normal distribution (mean 10 days, standard deviation of 2 days) and are at increased risk for 90 days after their initial course ends. Antibiotic risk exponentially decays from day 30 through day 90 with each day’s value equal to 97% of the previous day. This decay equation was selected because it produced the same level of CDI cases as simply leaving an agent at full increased risk for the first 60 days and then removing their risk on day 61 (which is what was originally implemented). By using this decay equation, we obtain a curve that produces similar CDI counts as that of the original 60-day method. The proportion of the antibiotic risk factor that is used for *x* days after an agent’s initial dose ends is shown in **Equation 1 (with figure).**

**Equation 1.**

$$p=2.4937e^{-0.03x}$$

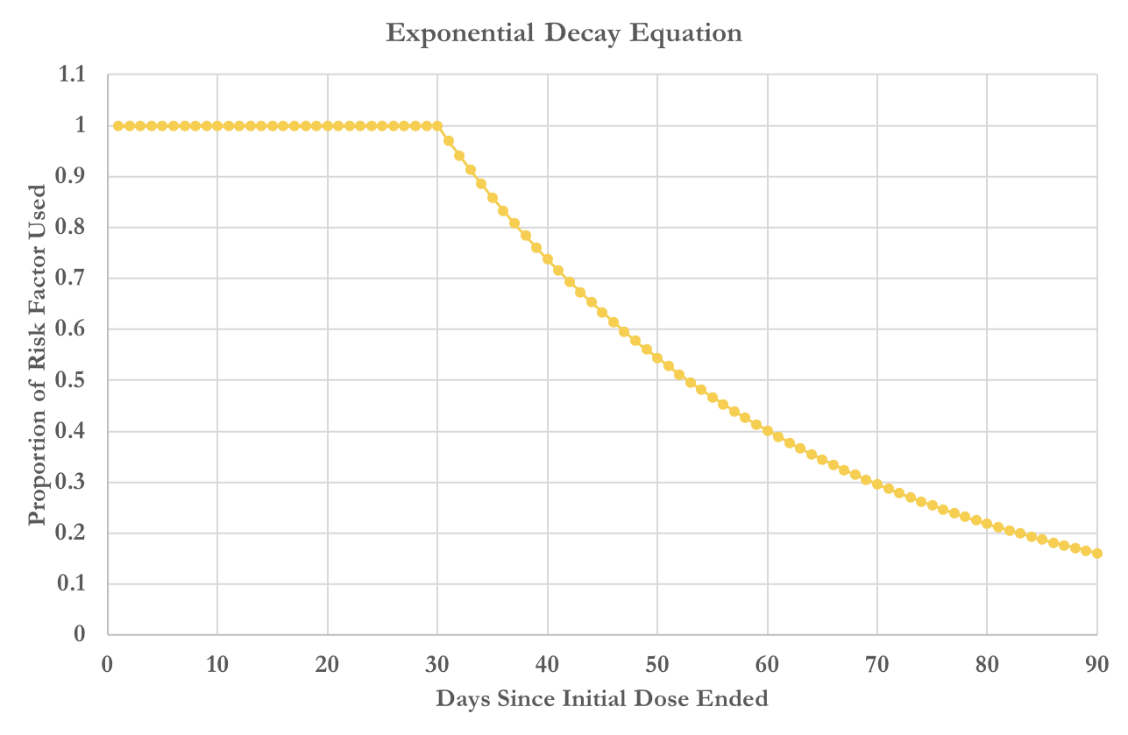


Once an agent’s current course of antibiotics ends, an agent can be assigned to a subsequent course. If assignment occurs during the residual risk period, the highest risk level of the two possible antibiotic risk levels is assigned to the agent.

Agents can recover from CDI and later return to the CDI state any number of times (i.e., recurrent CDI). Previous CDI occurrences and age ≥65 years increase an agent’s probability of recurrent CDI. Force of colonization is the probability that an uncolonized agent becomes asymptomatically colonized with *C. difficile* and is based on an agent’s location (STACH, LTACH, nursing home, community), including the number of patients at that location who are colonized and who have CDI. **Equation 2** shows an example of this calculation based on the STACH location [3].

**Equation 2.**

$$\lambda_{ST}=g(\beta_{S}\left( 1-\pi\right){CDI}_{ST}+\beta_{A}C_{ST})+(\pi\beta_{S}{CDI}_{ST}\left( 1-\epsilon\right))$$

$${CDI}_{ST}=\frac{\#CDI cases in STACH}{\#STACH patients}$$

$$C_{ST}=\frac{\#asymptomatic colonized STACH patients}{\#STACH patients}$$

$$\beta_{S}=Base CDI transmission rate within STACH$$

$$\beta_{A}=Base asymptomatic transmission rate within STACH$$

$$g=Overall hospital hygiene multiplier$$

$$\pi=Probability that patient with CDI is identifed and contact precautions employed$$

$$\epsilon=Effectiveness of contact precautions employed$$

In this way, the ABM framework of agent flow through the network is linked to the CDI disease model. The probability that an asymptomatically colonized patient develops CDI is determined by the agent’s antibiotic exposure status, age, presence of comorbidities, recent CDI count, and location [3].

We apply our CDI disease model based on the following agent attributes: (1) location state; (2) antibiotic state (i.e., currently exposed or currently not exposed) and the risk associated with an agent’s assigned antibiotic; (3) number of recurrent CDI events, defined as CDI event(s) within 14 days to 56 days of a previous CDI event (up to three; each additional recurrent CDI event will initiate a new 14−56 day window); (4) comorbidities (i.e., presence or absence); and (5) age [3, 17, 18]. We assign the presence of comorbidities to agents at model initialization, such that 23.74% of agents 50-64 years of age and 54.97% of agents ≥65 years of age have comorbidities [3]. We then adjust agents’ location transition probabilities, such that at any given time in the model, 55% of agents in healthcare facilities who are 50-64 years of age and 79% of agents in healthcare facilities who are ≥65 years of age will have comorbidities [3].

***iv. Initialization.*** The ABM is initialized with a geospatially explicit synthetic baseline population of NC based on the United States synthetic population (approximately 10 million agents) [19]. To initialize other agent variables (e.g., initial location, disease state), we merge initialization variables to the synthetic population using various data sources. First, agent locations are initialized using steady-state values for locations based on available data [11, 12]. Each STACH, LTACH, and nursing home location has an assigned number of beds, according to NC healthcare facility licensing data [13, 14]. On model initialization, 70% of STACH and nursing home beds are occupied and 90% of LTACH beds are occupied. To assign agents to each LTACH and nursing home, the ABM first finds all U.S. Census block groups that have that healthcare facility as its closest facility, based on U.S. Census block group centroid and facility addresses. Then, the ABM randomly assigns agents from those block groups to the healthcare facility. Only agents ≥65 years of age are eligible to move to a nursing home location. For each STACH, we calculate the number of agents that should come from each county, based on STACH discharge data [11, 12]. For agents who have not already been assigned an initialization location, the ABM then randomly selects patients from each county to fill each STACH. Agents are weighted by their age, such that 40% are <50 years of age, 20% are 50-64 years of age, and 40% are ≥65 years of age. Agents not assigned to any of the healthcare facilities will be in the community when the ABM simulation run begins.

Agents located in healthcare facility nodes are assigned a LOS based on the LOS distribution for that location. All agents are initialized without exposure to antibiotics and are either susceptible or asymptomatically colonized. Colonization prevalence is assigned randomly to each agent based on its location. The rate of colonization assignment is based on published data [3].

***v. Process overview and scheduling.*** A single step in a simulation run first updates the location of all agents, then the life status of agents, and finally the disease states of agents. Agent locations are updated based on location transition matrices parameterized with the following data sources: (1) aggregate hospital discharge data for NC [11, 12]; (2) published demographic characteristics of nursing home residents and LTACH patients [20, 21]; (3) NC licensed healthcare facility characteristics (i.e., capacity and occupancy) [13, 14, 20, 22, 23]; (4) de-identified patient-level hospital discharge data from UNC Health Care (July 1, 2016−June 30, 2017); and (5) CMS 2016 national patient-level fee-for-service claims data for CMS beneficiaries.

We assume that 2% of all location changes to STACHs (i.e., transfers) originating from non-UNC STACHs have UNC STACHs as their transfer location target based on subject matter expertise. Other transfer assumptions are as follows: (1) 90% of agents in a UNC STACH have another UNC STACH as their transfer location target while the other 10% transition to non-UNC STACHs; (2) 80% of agents in a large non-UNC STACH (who transfer to a non-UNC STACH) have another large non-UNC STACH as their transfer location target while the other 20% will transfer to a small non-UNC STACH; (3) 90% of agents in a small non-UNC STACH (who transfer to a non-UNC STACH) have a large non-UNC STACH as their transfer location target while the other 10% will transfer to a small non-UNC STACH. Of all agents moving from a nursing home to an STACH, 80% will return to the same nursing home when the STACH LOS ends [24]. Of all agents moving from a nursing home, 67.3% will return to the community (CMS 2016 national patient-level fee-for-service claims data for CMS beneficiaries). Finally, 9.4% of STACH admissions are, in fact, readmissions, in which an agent returns to the same STACH it had previously been admitted to.

Because the CDI state may lead to death, the life status of an agent is updated prior to an update to its disease state, simulating death from non-CDI related causes. Probabilities for transition to adjacent disease states depend on the current disease state of an agent (**S1 File Figure 2**). For recurrent CDI events, an agent first must leave the CDI state. Upon leaving the CDI state, agents are selected to return to the colonized state, which will eventually lead to a recurrent CDI, return to susceptible, or experience a CDI-associated death. An agent’s probability of returning to the colonized state is based on the agent’s number of recent CDI events. Upon returning to colonized, we use a CDI recurrence rate from the literature until the agent has another CDI event.

***vi. Input data.*** The model does not use input data to represent time-varying processes.

***vii. Agent interactions and organism transmission.*** Agents do not make explicit decisions. Agents do interact with their environment and, through their environment, interact with each other. **Equation 2** is based on the disease status of all the agents currently at a healthcare facility location node. Therefore, an agent’s probability of becoming colonized is influenced by the current status of the other agents at that same location.

***viii. Stochasticity.*** Stochasticity in the ABM results from random probabilities used to determine the following: (1) location changes, (2) disease state changes (including antibiotic exposure), (3) death, (4) agent initialization, and (5) LOS. Furthermore, after initial calibration is completed, model parameters are randomly selected from their distributions when completing additional model runs for analysis.

***viv. Model verification, validation, and calibration.*** Prior to calibrating the location and disease submodels we verified that simulation runs produced reasonable output of expected values. This was achieved by testing individual methods implemented in those submodels and running the entire model. Once verified, we calibrated the model according to specific targets informed by CMS 2016 national patient-level fee-for-service claims data for CMS beneficiaries (**S1 File Table 4**).

For the disease model, we verified that antibiotic exposure was occurring at reasonable rates by comparing the total number of antibiotic prescriptions at each location to published data. Subsequently, we calibrated colonization prevalence rates based on location [4]. Because the colonization rate is determined by the force of colonization equation, we created facility-specific multipliers to finely adjust colonization rate, rather than adjusting colonization clearance rates. Colonization is not a rare occurrence in the ABM, ranging from 6% to 15% prevalence at any given location. This facilitated us conducting numerous, small model runs to adjust each multiplier until all locations matched their colonization prevalence values.

For CDI we derived two sets of targets based on different CDI surveillance case definitions (**S1 File Table 5**). We used healthcare-associated CDI (HA-CDI) and community-associated CDI (CA-CDI) to calculate incidence per 100,000 persons [17, 25-27]. We used healthcare (hospital)-onset (HO-CDI) and community-onset (CO-CDI) to calculate incidence per 10,000 patient-days [28, 29]. Based on the available targets, we calibrated to the entire population rather than by age, presence of comorbidities, or other variables. To match CA-CDI and CO-CDI cases targets, the model randomly assigns CDI to a small number of agents.

To calibrate to these targets, we again created multipliers to adjust the transition to CDI, based on location. CDI incidence is a rare occurrence in the ABM, with only 5 cases per 10,000 patient-days to 10 cases per 10,000 patient-days. Therefore, conducting numerous, small runs for calibration was not feasible. Instead, we completed several larger runs using a random sample of 2,000,000 agents from the NC synthetic population. As CDI incidence fluctuates greatly across model runs, we updated multipliers until all targets were within 15% of a single model run.

**S1 File Table 1: Location model variables.**

| Variable | Description | Possible Values | Source |
| --- | --- | --- | --- |
| id | Identifier for each agent | [0, …) | model generated |
| age group | categorical age (years); fixed | [0(<50), 1(50-64), 2(65+)] | synthetic population |
| demographic id | a single value representing an agent’s age and North Carolina home county | [0, 1, 800] | synthetic population |
| community probability | daily probability that an agent leaves the community and moves to a healthcare facility | (0, 1) | *(11, 12, 20-22, 24),* de-identified patient-level hospital discharge data from UNC Health Care (July 1, 2016−June 30, 2017) |
| location status | current location of the agent | [0, …) | *model generated* |
| life status | current life status of the agent | [0 (dead), 1 (alive)] | *model generated* |
| current los | current length of stay for an agent; value of -1 if the agent is not at a healthcare facility | [-1, 1, …] | *(11, 12, 20-22);* de-identified patient-level hospital discharge data from UNC Health Care (July 1, 2016−June 30, 2017); Centers for Medicare & Medicaid Services 2016 national patient-level fee-for-service claims data |
| nursing home patient | binary flag signifying if the agent is currently in a nursing home | [0 (no), 1 (yes)] | model generated |
| leave facility day | day in the model that an agent will leave a healthcare facility; set when an agent changes location | [0, …) | model generated |
| death probability | daily probability that an agent dies; based on location and demographics | (0, 1) | *(25)* |

**S1 File Table 2:** ***Clostridioides difficile* infection (CDI) disease model and antibiotic parameters.**

| Parameter | Description | Values^1^ | Source |
| --- | --- | --- | --- |
| CDI Base Rate (Community) | Transition from colonized to CDI | 0.0000063 | *3* |
| CDI Base Rate (Hospital) | Transition from colonized to CDI | 0.00021 |  |
| CDI Base Rate (nursing home) | Transition from colonized to CDI | 0.0000860 |  |
| CDI Recovery^2^ | Transition from CDI to another state | 0.09426 |  |
| Recurrence^2^ | Transition from colonized to CDI for patients with a recent CDI | 0.1219 |  |
| Colonization Initialization (Community) | Colonization prevalence | 0.066 |  |
| Colonization Initialization (STACHs) | Colonization prevalence | 0.11 |  |
| Colonization Initialization (LTCFs) | Colonization prevalence | 0.148 |  |
| Colonization recurrence (1 recent CDI) | Colonization recurrence rate for agents with a recent CDI | 0.22 |  |
| Colonization recurrence (2 recent CDI) | Colonization recurrence rate for agents with a recent CDI | 0.33 |  |
| Colonization recurrence (3 recent CDI) | Colonization recurrence rate for agents with a recent CDI | 0.56 |  |
| Colonization clearance | Clearance to susceptible | 0.0198 |  |
| Base colonization rate (community) | Transition from susceptible to colonized | 0.00119928 |  |
| Base colonization rate (STACHs & LTACHs) | Used in the force of colonization equation | 0.022737516 |  |
| Base colonization rate (nursing homes) | Used in the force of colonization equation | 0.003693163 |  |
| Relative risk (concurrent conditions) | Increases risk of transitioning from colonized to CDI | 2.6 |  |
| Relative risk (age, 50-64) | Increases risk of transitioning from colonized to CDI | 2.2 |  |
| Relative risk (age, ≥65) | Increases risk of transitioning from colonized to CDI | 2.9 |  |
| Antibiotic prescribing rates  for non-network STACHs, LTACHs, nursing homes, and outpatient locations^3,4^ | for non-network STACHs, LTACHs, nursing homes, and outpatient locations | Non-network STACH: 0.37  LTACH: 0.37  Nursing home:  Outpatient, <50 years of age: 1.3x10^3^  Outpatient, 50-64 years of age: 1.4x10^3^  Outpatient, ≥65 years of age:1.7x10^3^ | *3* |
| Antibiotic course | - | 10 days (SD = 2 days) | Expert opinion |
| Antibiotic risk ratios | - | Low risk: 2  Moderate risk: 5  High risk: 12 | *3, 7, 29* |
| Baseline relative proportion of antibiotic use by risk class and location^3,4^ | By location | STACHs and LTACHs: proportion low risk = 0.4, proportion moderate risk = 0.3, proportion high risk = 0.3  Nursing homes and outpatient locations: proportion low risk = 0.1, proportion moderate risk = 0.6, proportion high risk = 0.3 | Patient-level data; expert opinion |
| ^1^Value rates are per day.  ^2^Adapted from the Source 3 daily rate to a daily probability.  ^3^Antibiotic exposure assigned to agents located in STACHs, LTACH, or nursing home nodes was conceptualized as the agent being “prescribed” the antibiotic at that healthcare facility.  ^4^Antibiotic exposure assigned to agents located in the community node was conceptualized as the agent being “prescribed” the antibiotic at an outpatient location. | | | |

**S1 File Table 3. Proportion of antibiotics administered by risk level and location.**

|  | **Low risk** | **Moderate risk** | **High risk** |
| --- | --- | --- | --- |
| **Outpatient*** | 0.1 | 0.6 | 0.3 |
| **LTACH** | 0.1 | 0.6 | 0.3 |
| **Nursing Home** | 0.1 | 0.6 | 0.3 |
| **STACH** | 0.4 | 0.3 | 0.3 |
| *Represents outpatient prescribing in the community node  STACH: short-term acute care hospital  LTACH: Long-term acute care hospital | | | |

**S1 File Table 4. Calibration target percentages for agent movement for the agent-based model.**

|  | ***To Location*** | | | | | | |
| --- | --- | --- | --- | --- | --- | --- | --- |
| ***From Location*** | **Community**  **(%)** | **UNC STACHs**  **(%)** | **≥400 beds, Non-UNC STACHs^1^**  **(%)** | **<400 beds, non-UNC STACHs^2^**  **(%)** | **LTACH**  **(%)** | **Nursing home^3^**  **(%)** | **Death** |
| **Community** | 0 | 11.8 | 41.1 | 37.4 | 0 | 9.8 | 0 |
| **UNC STACHs** | 83.2 | 2.9 | 0.3 | 0.05 | 0.2 | 11.2 | 2.2 |
| **≥400 beds, Non-UNC STACHs^1^** | 84.8 | 0.05 | 2.1 | 0.5 | 0.3 | 9.8 | 2.5 |
| **<400 beds,**  **Non-UNC STACHs^2^** | 81.4 | 0.08 | 3.5 | 0.4 | 0.2 | 12.7 | 1.9 |
| **LTACHs** | 47.0 | 0.9 | 3.3 | 2.9 | 0 | 44.9 | 1.0 |
| **Nursing homes^3^** | 57.2 | 3.7 | 12.7 | 11.4 | 0 | 0 | 15.0 |
| STACH: Short-term acute care hospital  UNC STACH: UNC Health Care STACH (combined across all 10 UNC STACHs in the agent-based model)  Non-UNC STACH: All other North Carolina STACHs  LTACH: Long-term acute care hospital (N=10)  ^1^Non-UNC STACHS with ≥400 beds (N=12)  ^2^Non-UNC STACHS with <400 beds (N=90)  ^3^Nursing homes (N=421) | | | | | | | |

**S1 File Table 5: Disease model *Clostridioides difficile* infection (CDI) calibration targets and achieved values.**

| **Metric** | **Target** | **Achieved value** | **Source** |
| --- | --- | --- | --- |
| Community-associated CDI | 30 cases to 120 cases per 100,000 agents | 78.9 cases per 100,000 agents | [17, 25-27] |
| Healthcare-associated CDI | 50 cases to 160 cases per 100,000 patients | 87.8 cases per 100,000 patients |  |
| Community-onset CDI for UNC STACHs | 12.12 cases per 10,000 patient-days | 12.3 cases per 10,000 patient-days | [28, 29] |
| Community-onset for non-UNC STACHs | 12.95 cases per 10,000 patient-days | 12.9 cases per 10,000 patient-days |  |
| Hospital-onset for UNC STACHs | 6.32 cases per 10,000 patient-days | 6.1 cases per 10,000 patient-days |  |
| Hospital-onset for non-UNC STACHs | 6.88 cases per 10,000 patient-days | 7.4 cases per 10,000 patient-days |  |
| UNC STACH: UNC Health Care short-term acute care hospital (combined across all 10 UNC STACHs in the agent-based model)  Non-UNC STACH: All other North Carolina STACHs | | | |

**S1 File Figure 1. Location entities and possible agent transitions in the agent-based model.**

| 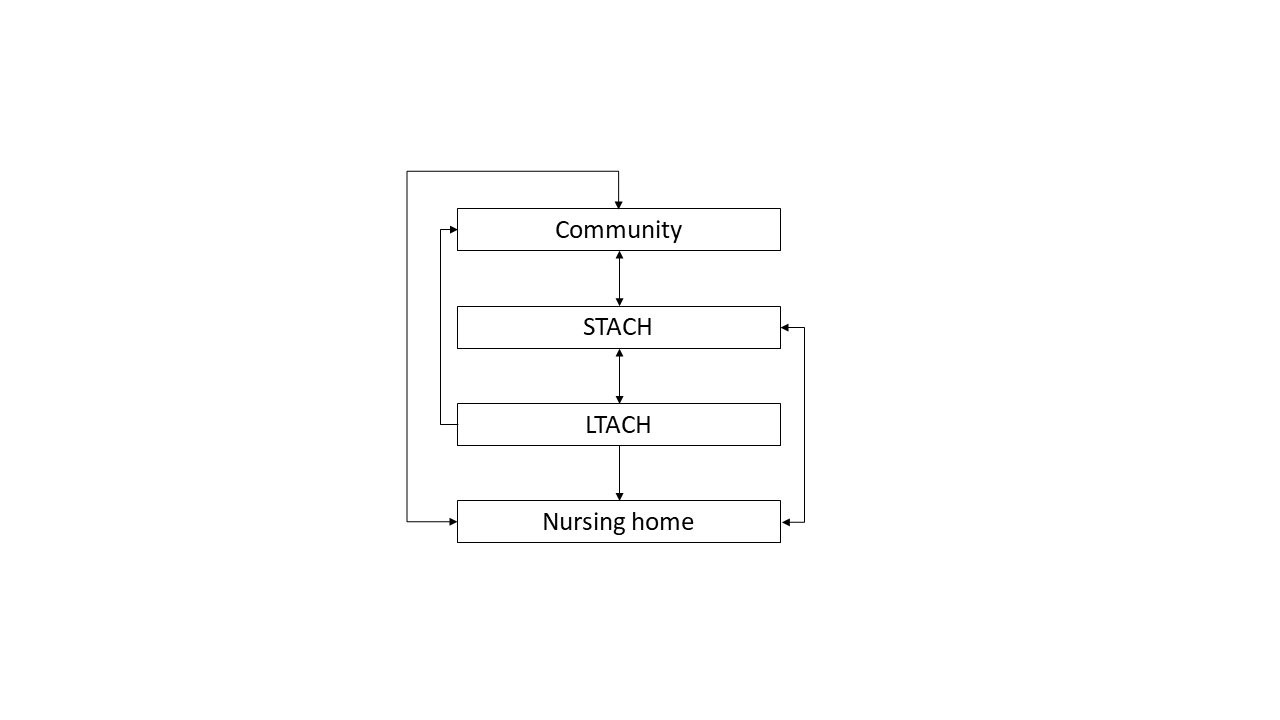 |
| --- |
| STACH: short-term acute care hospital; LTACH: long-term acute care hospital; |

**S1 File Figure 2. *Clostridioides difficile (C. difficile)* disease model diagram with parameters.**

| **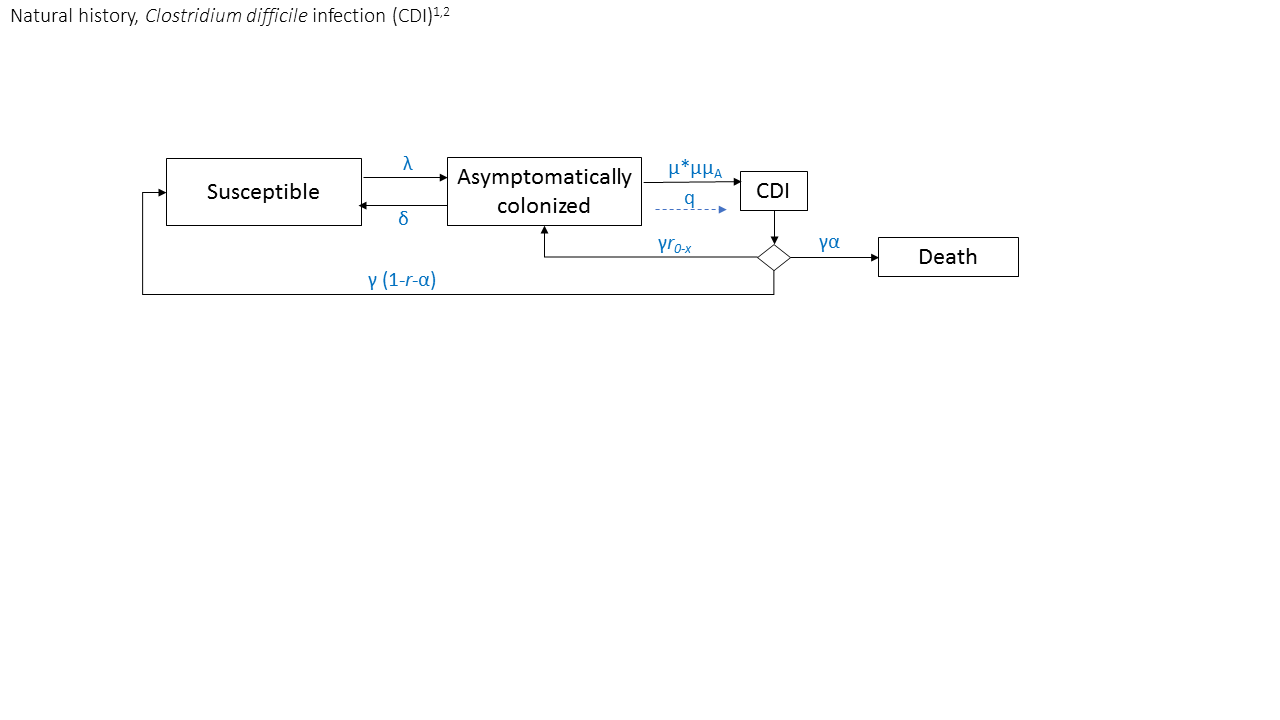** |
| --- |
| \| Parameter \| Description \| Source \| \| --- \| --- \| --- \| \| λ \| Force of colonization by location (community, STACH, LTACH, nursing home) \| 3 \| \| δ \| Spontaneous clearance of *C. difficile* colonization \| 3 \| \| μ \| Relative risk for developing CDI (μ_A_ while receiving antimicrobial drugs; μ50 among persons 50–65 years of age vs. those <50 years of age; μ65 among persons >65 years of age vs. those <50 years of age) \| 3 \| \| q \| Probability that recovered patients show recurrence \| 3 \| \| r \| Probability that a patient recovering from primary CDI will have at least 1 recurrence \| 3 \| \| γ \| Probability of recovery from CDI \| 3 \| \| α \| All-cause CDI mortality \| 3 \| |
| CDI: *Clostridioides* *difficile* infection  STACH: short-term acute care hospital  LTACH: long-term acute care hospital |

**References**

1. Grimm V, Berger U, Bastiansen F, Eliassen S, et al. A standard protocol for describing individual-based and agent-based models. Ecological Modeling. 2006;198: 115-126.

2. Grimm V, Berger U, DeAngelis DL, Polhill JG, Giske J, Railsback SF. The ODD protocol: A review and first update. Ecological Modelling. 2010;221(23): 2760-2768. doi: 10.1016/j.ecolmodel.2010.08.019

3. Durham DP, Olsen MA, Dubberke ER, Galvani AP, Townsend JP. Quantifying transmission of *Clostridium difficile* within and outside healthcare settings. Emerg Infect Dis. 2016 Apr;22(4): 608-616. doi: 10.3201/eid2204.150455

4. Dubberke ER, Yan Y, Reske KA, Butler AM, Doherty J, Pham V, et al. Development and validation of a *Clostridium difficile* infection risk prediction model. Infect Control Hosp Epidemiol. 2011 Apr;32(4): 360-366. doi: 10.1086/658944

5. Loo VG, Poirier L, Miller MA, Oughton M, Libman MD, Michaud S, et al. A predominantly clonal multi-institutional outbreak of *Clostridium difficile*-associated diarrhea with high morbidity and mortality. N Engl J Med. 2005 Dec 8;353(23): 2442-2449. doi: 10.1056/NEJMoa051639

6. Hensgens MP, Goorhuis A, Dekkers OM, Kuijper EJ. Time interval of increased risk for *Clostridium difficile* infection after exposure to antibiotics. J Antimicrob Chemother. 2012 Mar;67(3): 742-748. doi: 10.1093/jac/dkr508

7. Brown KA, Fisman DN, Moineddin R, Daneman N. The magnitude and duration of *Clostridium difficile* infection risk associated with antibiotic therapy: a hospital cohort study. PLoS One. 2014;9(8): e105454. doi: 10.1371/journal.pone.0105454

8. Riggs MM, Sethi AK, Zabarsky TF, Eckstein EC, Jump RL, Donskey CJ. Asymptomatic carriers are a potential source for transmission of epidemic and nonepidemic *Clostridium difficile* strains among long-term care facility residents. Clin Infect Dis. 2007 Oct 15;45(8): 992-998. doi: 10.1086/521854

9. McDonald LC, Gerding DN, Johnson S, Bakken JS, Carroll KC, Coffin SE, et al. Clinical practice guidelines for *Clostridium difficile* infection in adults and children: 2017 update by the Infectious Diseases Society of America (IDSA) and Society for Healthcare Epidemiology of America (SHEA). Clin Infect Dis. 2018 Mar 19;66(7): e1-e48. doi: 10.1093/cid/cix1085

10. UNC Health Care. Preparing for the future of health care. UNC health care 2017 annual report. 2017 [cited January 21 2019]. Chapel Hill, NC: UNC Health Care. Available: <https://www.unchealthcare.org/app/files/public/10436/PDF-MedCtr-Annual-Report-2017.pdf>.

11. Cecil G. Sheps Center for Health Services Research, University of North Carolina at Chapel Hill. Short term acute care hospital discharge data - patient characteristics. Summary data for all hospitals (October 1, 2016 through September 30, 2017). 2017 [cited October 2019]. Chapel Hill, NC: Cecil G. Sheps Center for Health Services Research,, University of North Carolina at Chapel Hill. Available: <https://www.shepscenter.unc.edu/wp-content/uploads/2019/04/ptchar_all_and_by_hosp_2017_and.pdf>.

12. Cecil G. Sheps Center for Health Services Research, University of North Carolina at Chapel Hill. Patient county of residence by hospital (October 1, 2016 through September 30, 2017). 2017 [cited October ` 2019]. Chapel Hill, NC: Cecil G. Sheps Center for Health Services Research, University of North Carolina at Chapel Hill. Available: <https://www.shepscenter.unc.edu/wp-content/uploads/2019/04/ptorg_pt_res_by_hosp_2017.pdf>.

13. NC Department of Health and Human Services, Division of Health Service Regulation. Hospitals by county 2018. 2018 March 5 [cited March 8 2018]. Raleigh, NC: Department of Health and Human Services - Division of Health Service Regulation. Available: <https://www2.ncdhhs.gov/dhsr/data/hllistco.pdf>.

14. NC Department of Health and Human Services, Division of Health Service Regulation. Nursing homes by county 2018. 2018 March 5 [cited March 5 2018]. Raleigh, NC: Department of Health and Human Services - Division of Health Service Regulation. Available: <https://www2.ncdhhs.gov/dhsr/data/nhlist_co.pdf>.

15. Dubberke ER, Butler AM, Reske KA, Agniel D, Olsen MA, D'Angelo G, et al. Attributable outcomes of endemic Clostridium difficile-associated disease in nonsurgical patients. Emerg Infect Dis. 2008 Jul;14(7): 1031-1038. doi: 10.3201/eid1407.070867

16. Kyne L, Hamel MB, Polavaram R, Kelly CP. Health care costs and mortality associated with nosocomial diarrhea due to Clostridium difficile. Clin Infect Dis. 2002 Feb 1;34(3): 346-353. doi: 10.1086/338260

17. Lessa FC, Mu Y, Bamberg WM, Beldavs ZG, Dumyati GK, Dunn JR, et al. Burden of *Clostridium difficile* infection in the United States. N Engl J Med. 2015 Feb 26;372(9): 825-834. doi: 10.1056/NEJMoa1408913

18. Figueroa I, Johnson S, Sambol SP, Goldstein EJC, Citron DM, Gerding DN. Relapse versus reinfection: recurrent *Clostridium difficile* infection following treatment with fidaxomicin or vancomycin. Clin Infect Dis. 2012;55(Suppl 2): S104–109.

19. Wheaton WD, Cajka JC, Chasteen BM, Wagener DK, Cooley PC, Ganapathi L, et al. Synthesized population databases: a US geospatial database for agent-based models. Methods Rep RTI Press. 2009 May 1;2009(10): 905. doi: 10.3768/rtipress.2009.mr.0010.0905

20. Centers for Medicare & Medicaid Services. Nursing home data compendium 2015 edition. Baltimore, MD: Centers for Medicare & Medicaid Services (CMS); 2015.

21. Kahn JM, Barnato AE, Lave JR, Pike F, Weissfeld LA, Le TQ, et al. A comparison of free-standing versus co-located long-term acute care hospitals. PLoS One. 2015;10(10): e0139742. doi: 10.1371/journal.pone.0139742

22. MedPac (2016) Report to the Congress. . Medicare Payment Policy. Washington, DC: The Medicare Payment Advisory Commission (MedPAC). pp. 288.

23. NC Department of Health and Human Services. North Carolina Health Statistics Pocket Guide. 2015 February 28, 2017 [cited January 21 2019]. Raleigh, NC: NC Division of Public Health. Available: <https://schs.dph.ncdhhs.gov/data/pocketguide/2015/>.

24. Toth DJA, Khader K, Slayton RB, Kallen AJ, Gundlapalli AV, O'Hagan JJ, et al. The potential for interventions in a long-term acute care hospital to reduce transmission of carbapenem-resistant enterobacteriaceae in affiliated healthcare facilities. Clin Infect Dis. 2017 Aug 15;65(4): 581-587. doi: 10.1093/cid/cix370

25. Centers for Disease Control and Prevention. Healthcare-associated infections - community interface (HAIC). 2016 annual report. January 2 [cited July 31 2019]. Atlanta, GA: CDC. Available: <https://www.cdc.gov/hai/eip/Annual-CDI-Report-2016.html>.

26. Centers for Disease Control and Prevention. Healthcare-associated infections - community interface (HAIC). Clostridioides difficiile infection (CDI) tracking. January 2 [cited July 31 2019]. Atlanta, GA: CDC. Available: <https://www.cdc.gov/hai/eip/cdiff-tracking.html?CDC_AA_refVal=https%3A%2F%2Fwww.cdc.gov%2Fhai%2Feip%2Fclostridium-difficile.html>.

27. Kutty PK, Woods CW, Sena AC, Benoit SR, Naggie S, Frederick J, et al. Risk factors for and estimated incidence of community-associated *Clostridium difficile* infection, North Carolina, USA. Emerg Infect Dis. 2010 Feb;16(2): 197-204. doi: 10.3201/eid1602.090953

28. Centers for Disease Control and Prevention. Multidrug-resistant organism & clostridioides difficile infection (MDRO/CDI) module. 2019 [cited July 31 2019]. Atlanta, GA: CDC. Available: <https://www.cdc.gov/nhsn/PDFs/pscManual/12pscMDRO_CDADcurrent.pdf>.

29. Slimings C, Riley TV. Antibiotics and hospital-acquired Clostridium difficile infection: update of systematic review and meta-analysis. J Antimicrob Chemother. 2014 Apr;69(4): 881-891. doi: 10.1093/jac/dkt477
